# Statins as Potential Preventative Treatment of ETX and Multiple Pore-Forming Toxin-Induced Diseases

**DOI:** 10.3390/ijms24065414

**Published:** 2023-03-12

**Authors:** Jing Huang, Baohua Zhao, Tingting Liu, Lin Kang, Jiaxin Li, Zishuo Guo, Ming Chen, Shan Gao, Jing Wang, Yanwei Li, Jinglin Wang, Wenwen Xin

**Affiliations:** 1 Life Science Institute, Hebei Normal University, Shijiazhuang 050024, China; 2 State Key Laboratory of Pathogen and Biosecurity, Beijing Institute of Microbiology and Epidemiology, Academy of Military Medical Sciences (AMMS), Beijing 100071, China

**Keywords:** statins, *Clostridium perfringens* ε-toxin, Zaragozic acid, therapy, pore-formation

## Abstract

Epsilon toxin (ETX), produced by type B and D strains of *Clostridium perfringens*, can cause fatal enterotoxaemia in ruminant animals, particularly sheep, cattle, and goats. Previous studies show that the cytotoxicity of ETX is dependent on the integrity of lipid rafts, the maintenance of which is ensured by cholesterol. Zaragozic acid (ZA) is a statin drug that reduces the synthesis of squalene, which is responsible for cholesterol synthesis. In this study, ZA significantly reduced the toxicity of ETX in Madin–Darby canine kidney (MDCK) cells. We show that ZA does not affect the binding of ETX to MDCK cells, but propidium iodide staining (PI) and Western blotting confirmed that ZA significantly disrupts the ability of ETX to form pores or oligomers in MDCK cells. Additionally, ZA decreased the phosphatidylserine exposure on the plasma membrane and increased the Ca^2+^ influx of the cells. Results of density gradient centrifugation suggest that ZA decreased the number of lipid rafts in MDCK membranes, which probably contributed to the attenuation of pore-formation. Moreover, ZA protected mice against ETX in vivo. All mice pre-treated with ZA for 48 h before exposure to an absolute lethal dose of ETX (6400 ng/kg) survived. In summary, these findings provide an innovative method to prevent ETX intoxication. Considering many pore-forming toxins require lipid rafts, we tested and found ZA also inhibited the toxicity of other toxins such as *Clostridium perfringens* Net B and β-toxin (CPB) and *Staphylococcus aureus* α-hemolysin (Hla). We expect ZA can thus be developed as a broad-spectrum medicine for the treatment of multiple toxins. In addition, other statins, such as lovastatin (LO), also reduced the toxicity of ETX. These findings indicate that statin medicines are potential candidates for preventing and treating multiple toxin-induced diseases.

## 1. Introduction

*Clostridium perfringens* is widespread in nature, one of the world’s most common pathogens and responsible for numerous zoonotic diseases [1]. *C*. *perfringens* produces at least 17 exotoxins [2] and is divided into 7 toxinotypes (types A–G) based on its 6 main lethal toxins (α, β, ε, ι, CPE, and Net B) [3,4]. Epsilon toxin (ETX) is secreted by type B and D strains of *C*. *perfringens* as an inactive prototoxin that is activated after the removal of the N- and C-terminal peptides by proteases [5]. ETX causes rapidly fatal enterotoxaemia in ruminant livestock, such as sheep, goats, and cattle, leading to large losses in animal husbandry annually [6]. When injected into rats, the toxin accumulates mostly in the kidneys and brain, resulting in injury to the blood–brain barrier and possible death [7,8]. ETX is the third most potent biological toxin after botulinum neurotoxins and tetanus toxin [9], with a 50% lethal dose (LD_50_) of 65–110 ng/kg in mice [5,10], and is consequently classified as a category B biological agent by the United States Centers for Disease Control and Prevention [11].

Various cell lines are sensitive to ETX, including Madin–Darby canine kidney (MDCK) cells [12], murine renal cortical collecting duct principal (mpkCCDcl4) cells [13], human leiomyoblastoma (G-402) cells [14], Fischer rat thyroid cells (FRT) [15], and human renal adenocarcinoma (ACHN) cells [9,16]. Among these, MDCK cells are the most sensitive to ETX and have typically been used in previous ETX studies. As a pore-forming toxin, ETX binds to its putative receptor and forms a heptameric membrane complex on the MDCK cell membrane [17]. This leads to membrane permeabilization, which, in turn, results in a rapid decrease in intracellular K^+^, a rapid increase in Na^+^ and Cl^−^, and a delayed increase in Ca^2+^ [18,19]. Eventually, cells are killed by morphological changes, including swelling and the formation of membrane blebs [20]. The CT_50_ (the dose needed to kill 50% of cells) of ETX in MDCK cells is 15 ng/mL [21].

The interaction between ETX and host cells requires an appropriate lipid environment. For example, ETX preferentially forms a heptameric pore within the detergent-resistant membranes (DRM) of MDCK cells, which are cholesterol- and glycosphingolipid-enriched microdomains [22]. Moreover, the removal of cholesterol by methyl-β-cyclodextrin (MbCD) impairs ETX binding and complex formation [13,22,23].

Many vaccine candidates are effective in protecting against ETX-induced diseases [19,24,25]. However, few options for overcoming ETX toxicity have been reported. One of these is neutralizing antibodies against ETX [26]. Two small-molecule inhibitors of ETX cytotoxicity have been identified as potential treatments [27]. However, given the potential use of ETX in bioterrorism, alternative countermeasures that inhibit the activity of the toxin are needed. A novel approach to drug development is to place the focus on the affected host cells instead of targeting the causative agent. The numerous host factors involved in ETX-induced cytotoxicity provide potential targets to block the toxic effects of ETX. Antagonists or agonists of these targets can potentially be used for new drug design and treating ETX poisoning [28]. Cytotoxicity of ETX depends on the integrity of lipid rafts, and cholesterol plays a vital role in maintaining the integrity of lipid rafts. This led us to speculate that lipid-lowering drugs may work as inhibitors of ETX cytotoxicity. Zaragozic acid (ZA) is a statin drug that inhibits the synthesis of squalene, which is responsible for cholesterol synthesis [29].

In this study, we investigate whether ZA can reduce the toxic effects of ETX on MDCK cells and explore the possibility of ZA as a candidate treatment for the toxic effects of ETX. We also explored whether lovastatin (LO), another statin medicine, could protect against ETX and the potential of ZA to treat other pore-forming toxins.

## 2. Results

### 2.1. ZA Reduced Toxicity of ETX on MDCK Cells

**Figure 1:**
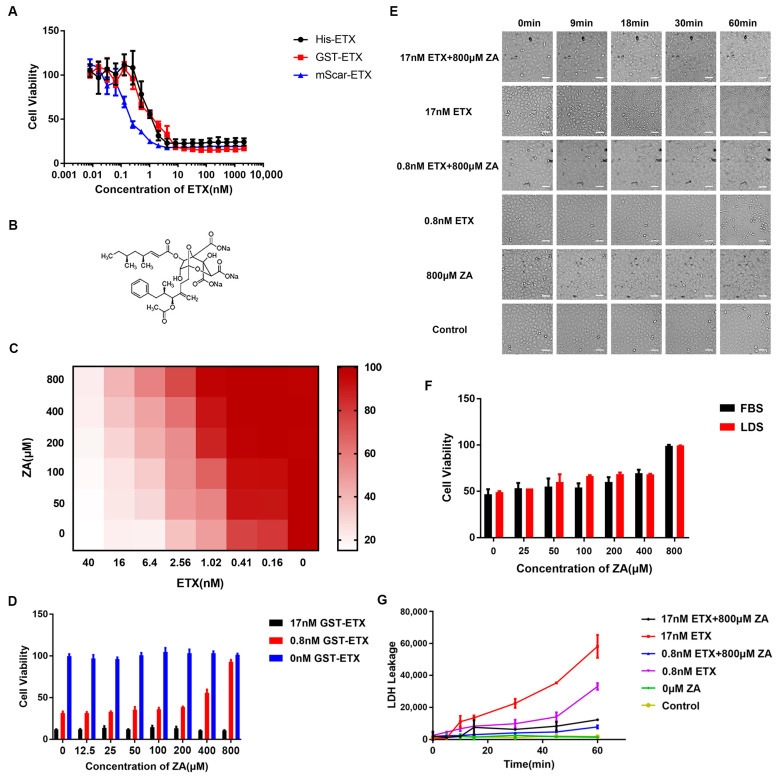
Cytotoxicity of Epslion toxin (ETX) toward Madin–Darby canine kidney (MDCK) cells was inhibited by Zaragozic acid A trisodium salt (ZA). (**A**) Toxicities of ETX with different tags are similar. (**B**) Chemical structure of ZA. (**C**,**D**) Cell viability (from MTS assays) in MDCK cells exposed to increasing concentrations of ZA for 30 min and then incubated with different concentrations of ETX with GST tag (GST-ETX) for 1 h. (**E**) MDCK cells were preincubated with ZA for 30 min and incubated with ETX and continuously observed for 1 h. Scale bar: 50 μm. (**F**) The MDCK cells were cultured for 24 h with DMEM supplemented with 10% fetal bovine serum (FBS) or 10% lipid-depleted fetal bovine serum (LDS) and then treated with ZA prior to exposure to 0.8 nM ETX. (**G**) MDCK cells treated with 800 µM ZA for 30 min were exposed to ETX for 0, 5, 10, 15, 30, 45, and 60 min. Then, the Lactic Dehydrogenase (LDH) released from cells was measured using the LDH-Glo^TM^ Cytotoxicity Assay Kit.

ETX with different tags (GST, 6×His, and mScar) did not differ significantly in toxicity (Figure 1A). The ETX (GST-ETX and His-ETX) dose needed to kill ~50% of MDCK cells (CT_50_) was 0.8 nM, and the CT_50_ of mScar-ETX was 0.4 nM, in agreement with the reported CT_50_ of 0.5 to 10 nM for the active wild-type ETX [13,20,21,26,30]. mScar-ETX exhibits stronger toxicity than ETX with other tags, probably because the mScar tag could enhance the stability of ETX. To investigate whether ZA (Figure 1B) could influence the cytotoxicity of ETX toward MDCK cells, cells were treated with a series of concentrations of ZA and then incubated with GST-ETX. A ZA concentration as high as 800 µM showed no cytotoxicity to MDCK cells. ZA significantly reduced the toxicity of 0.8 nM GST-ETX in a concentration-dependent manner, such that 800 µM ZA nearly abolished the toxicity of 0.8 nM GST-ETX (Figure 1C,D). The EC_50_ (concentration for 50% of the maximal effect) value for ZA was 151.8 μM. However, ZA had no obvious effect on MDCK cells treated with a high concentration (17 nM) of GST-ETX.

Morphological changes in MDCK cells exposed to ETX with or without ZA were observed (Figure 1E). When the concentration of ETX was 17 nM, the MDCK cells displayed shrinkage at 9 min, and at 18 min, cells were completely lysed. In contrast, the changes of MDCK cells exposed to 0.8 nM ETX were much slower; cells started to shrink at ~30 min and were completely lysed at ~1 h. In the presence of 800 µM ZA, 17 nM ETX leads to cell shrinkage at 18 min and total cell lysis by ~30 min, while the morphology of MDCK cells exposed to 0.8 nM ETX did not change compared to the control group. In order to exclude the influence of the cholesterol in the medium, the MTS assay was used to test the effect of ZA against 0.8 nM ETX in MDCK cells cultured in medium supplemented with 10% fetal bovine serum (FBS) or 10% lipid-depleted fetal bovine serum (LDS). The results showed no significant difference between the two groups (Figure 1F). Measures of Lactic Dehydrogenase (LDH) leakage revealed that ETX results in the release of intracellular LDH to the extracellular matrix (Figure 1G). However, the LDH released from ZA-treated cells was greatly reduced, which also suggests that ZA can reduce the cytotoxicity of ETX on MDCK cells.

### 2.2. ETX Binds to MDCK Cells Exposed to ZA

ETX initiates various steps that induce the death of cells. Specifically, ETX first binds to a putative receptor on the surface of the cell membrane and then forms pores in the regions of DEM [22,31]. Subsequently, ions permeate through the cell membrane and phosphatidylserine exposure on the plasma membrane occurs [32]. The promising effect of ZA against ETX led us to explore which of these steps was obstructed by ZA.

**Figure 2:**
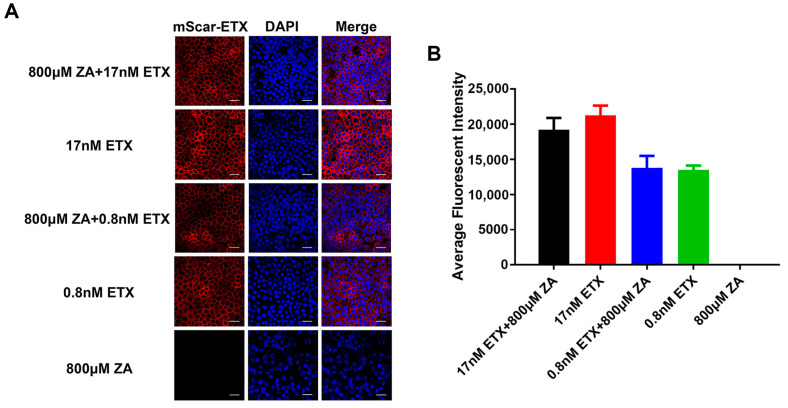
ZA does not inhibit binding of ETX to the membrane of MDCK cells. MDCK cells were incubated with ZA for 30 min and then treated with mScar-ETX for 1 h. (**A**) After staining with DAPI (blue), the cells were observed using confocal microscopy. Scale bar: 50 μm. (**B**) The average fluorescent intensity of ETX for different groups.

Binding to host cells is the first step by which ETX acts to induce cell death. Therefore, a binding assay was performed to study whether ZA affects the binding of ETX to MDCK cells. Confocal microscopy images revealed peripheral staining of mScar-ETX in MDCK cells, indicating that ETX was bound to the plasma membrane (Figure 2A). The average fluorescent intensity of MDCK cells first exposed to ZA was similar to cells not exposed to ZA (Figure 2B), indicating that ZA did not prevent the binding of ETX to MDCK cells.

### 2.3. ZA Reduces the Heptamer Formation on MDCK Cells

**Figure 3:**
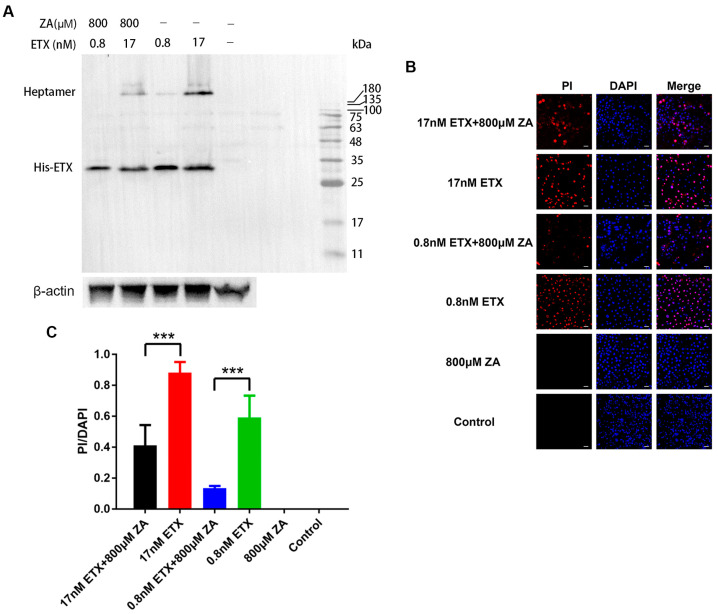
ZA reduces the formation of ETX heptamers and inhibits ETX-induced pore-formation in MDCK cells. (**A**) MDCK cells were incubated with ZA for 30 min and then treated with His-ETX for 1 h. Western blot analysis of cell lysates using anti-His revealed oligomeric complexes (~224 kDa) and monomeric forms of His-ETX (~32 kDa). Untreated MDCK cells were used as a control. (**B**) MDCK cells were cultured and incubated with 800 µM ZA for 30 min. Then, the medium was mixed with GST-ETX and propidium iodide staining (PI), followed by incubation for 1 h. After washing three times with PBS, the samples were stained with DAPI and observed using confocal microscopy. Scale bar: 50 μm. (**C**) The percent of PI-positive cells (PI/DAPI) with cells in different groups. *** *p* < 0.001.

To assess whether ZA reduces the capacity of ETX to form heptamers in MDCK cells, immunoblotting was performed. A high-molecular-weight band of ~224 kDa (the expected molecular weight of the heptameric complex) was observed in MDCK cells exposed to ETX alone. In cells exposed to ZA prior to ETX exposure, the grayscale of the heptamer band was significantly weakened at both concentrations of ETX tested (Figure 3A and Figure S1). This suggests ZA inhibits the formation of the heptamer. The monomeric ETX (band of ~32 kDa) was not obviously affected by ZA, indicating that the binding of ETX on cells was not affected by ZA, in agreement with the binding assay results above (Figure 2).

### 2.4. ZA Inhibits the ETX-Induced Pore Formation on MDCK Cells

Previous studies have shown that ETX forms heptamers on MDCK cells, followed by the formation of β-barrel heptameric transmembrane pores [20,33]. To assess whether ZA inhibits such pore formation, we performed a pore-forming assay using propidium iodide staining (PI) staining, which has been demonstrated to cross these ETX-formed pores. As shown in Figure 3B, PI entered cells treated with 0.8 nM ETX and stained the nucleus, while cells treated with ZA had decreased entry of PI into cells. At an ETX concentration of 17 nM, PI entry into the MDCK cells in the presence of ZA was also reduced, although to a lesser extent (Figure 3C), suggesting that ZA can also disturb pore formation in MDCK cells at a high ETX concentration.

### 2.5. ZA Prevents Toxin-Induced Phosphatidylserine Exposure

**Figure 4:**
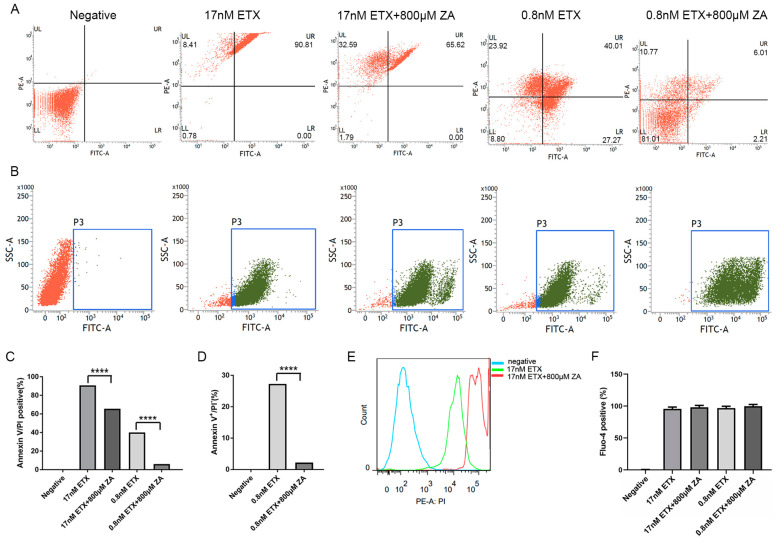
ZA prevents toxin-induced PS exposure but strengthens Ca^2+^ influx of MDCK cells. (**A**) MDCK cells were exposed to ZA for 30 min, followed by the addition of different concentrations of GST-ETX (17 and 0.8 nM) for 1 h at 37 °C. Then, cells stained by PI (PE-A channel) and annexin V (FITC channel) were analyzed on a flow cytometer. (**B**) The influx of Ca^2+^ in MDCK cells was measured in a flow cytometer by incubation with fluo-4 AM (5 μM). (**C**) Annexin V and PI-positive cells were counted and normalized to the number of total cells. (**D**) Annexin-V-positive and PI-negative cells were counted and normalized to the number of total cells. (**E**) Frequency curve of Figure 4A. (**F**) Fluo-4-positive cells were counted and normalized to the number of total cells. **** *p* < 0.0001.

ETX was previously reported to trigger phosphatidylserine (PS) exposure on the plasma membrane of human erythrocytes [32], an indicator of apoptosis [34]. Similarly, ETX triggered PS exposure on 90.81% of MDCK cells exposed to 17 nM ETX and 67.28% of MDCK cells exposed to 0.8 nM ETX (Figure 4A). ZA significantly decreased the percentage of cells with PS exposure to 65.62% with 17 nM ETX and 8.22% with 0.8 nM ETX (Figure 4A). These results indicate that ZA decreases ETX-induced apoptosis (Figure 4D) and the death of cells (Figure 4C). We also measured the proportion of cells positive for PI staining. In the group with 17 nM ETX, 99.22% of cells were stained with PI; while ZA had no effect on the percentage of cells stained, it did reduce staining intensity (Figure 4A,E). In the 0.8 nM ETX group, ZA decreased the proportion of PI-stained cells from 63.93% to 16.78% (Figure 4A). These results are consistent with the results from our pore formation assay above (Figure 3). The protective effect of ZA on cells is associated with the concentration of ETX. The higher the concentration of ETX, the lower the protective effect of ZA on cells.

### 2.6. ZA Strengthens Ca^2+^ Influx of MDCK Cells Treated with ETX

To assess whether ZA can prevent the toxin-induced Ca^2+^ influx in MDCK cells, intracellular Ca^2+^ concentrations of cells were measured (Figure 4B,F). ETX exposure increased intracellular Ca^2+^ concentration of MDCK cells; 95.6% of cells in the 17 nM group and 96.8% of cells in the 0.8 nM group showed a clear Ca^2+^ influx. Surprisingly, ZA further increased the proportion of cells with Ca^2+^ influx (Figure 4B,F). In particular, the fluorescence intensity increased (Figure 4B, Figures S2 and S4), indicating that ZA increased the Ca^2+^ influx of MDCK cells treated with ETX. We also tested whether ZA could strengthen the Ca^2+^ influx of MDCK cells without ETX and found that ZA alone induces potent Ca^2+^ influx in MDCK cells (Figures S3 and S4). However, the role of Ca^2+^ influx in the cytotoxicity of ETX has yet to be precisely determined. A study indicated that the increments of the intracellular Ca^2+^ concentration reduced PS exposure and protected the cells [35]. Therefore, ZA-induced Ca^2+^ influx in MDCK cells probably has a positive effect on cells.

### 2.7. ZA Protects Mice from Death by ETX

**Figure 5:**
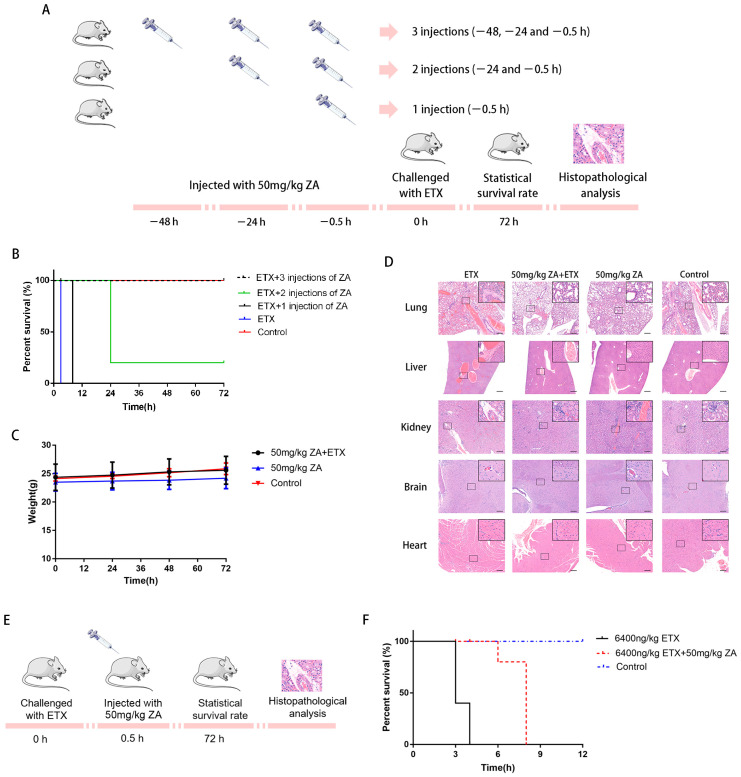
ZA can protect mice against ETX. (**A**) Mice were injected with 6400 ng/kg ETX 30 min after the administration of a first, second, or third dose of ZA. Mice in the control group were injected with PBS. The survival rate (**B**) and the weight (**C**) of mice after injections with ZA for 2 days, followed by ETX challenge and then observed for 72 h. (**D**) Sections were made from various organs of the challenged mice, stained with hematoxylin and eosin (H & E). Photographs of the sections were taken using a bright field microscope with a digital camera. Scale bar: 100 μm. (**E**,**F**) Mice were injected with 50 mg/kg ZA 30 min after being challenged with 6400 ng/kg ETX.

To see whether ZA can prevent the toxin-induced death of animals, BALB/c mice were given an intraperitoneal injection of ZA (50, 10, 2, and 0.4 mg/kg/day) (Figure S5) and then challenged with an absolute lethal dose of ETX (6400 ng/kg) (Figure S6) on time 0; 3 dosing groups were tested: 3 injections (−48, −24 and −0.5 h), 2 injections (−24 and −0.5 h), and 1 injection (−0.5 h) (Figure 5A). ZA significantly improved the survival rate of mice challenged with ETX (Figure 5B). All control mice challenged with an absolute lethal dose of ETX died within 3 h. In contrast, all mice injected with ZA three times and then challenged with ETX survived. Though the mice injected with ZA for 2 injections or 1 injection before the challenge with ETX died (4/5 dead for 2 injections and 5/5 dead for 1 injection), ZA significantly extended the survival time (Figure 5B). In addition, the weight of surviving mice at different time points suggests that ZA protected mice and kept them in healthy living conditions (Figure 5C). We performed histopathological analysis on the organs (liver, kidney, lung, brain, and heart) of all mice 3 days after the ETX injection. In mice that died from ETX, obvious hemorrhage was present in the liver, kidney, and lung, while edema was present in the lung, liver, kidney, and brain of the ETX-only treated group. However, mice in the ZA-treated group (three injections) followed by the ETX challenge did not show hemorrhage or edema in the organs (Figure 5D). In addition, no histopathological changes were observed in the group injected with ZA alone or the group injected with PBS (Figure 5D). These results indicate that ZA can protect mice against ETX. Finally, we tested the ability of ZA injected 30 min after the ETX challenge (Figure 5E) and found that ZA significantly extended the survival time (from 4 to 8 h), although all mice died (Figure 5F).

**Figure 6:**
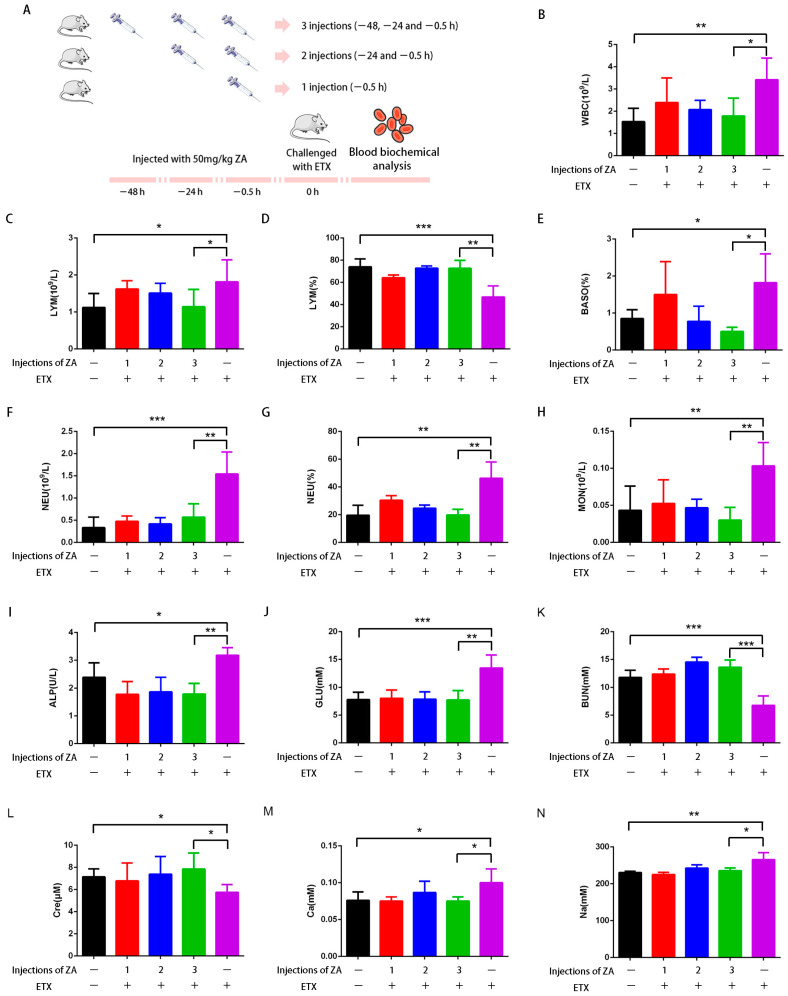
ZA can effectively protect mice from the toxicity of ETX. (**A**) Mice were injected with 50 mg/kg/day ZA for 3 injections (−48, −24, and −0.5 h), 2 injections (−24 and −0.5 h) or 1 injection (−0.5 h) only, then challenged with 6400 ng/kg ETX at time 0. The blood of mice was collected, and blood parameters were examined. (**B**) White blood cells (WBC). (**C**) Number of lymphocytes (LYM). (**D**) Lymphocyte ratio (LYM%). (**E**) Basophils ratio (BASO%). (**F**) Number of neutrophils (NEU). (**G**) Neutrophil ratio (NEU%). (**H**) Number of monocytes (MON). (**I**) Alkaline phosphatase (ALP). (**J**) Glucose (GLU). (**K**) Urea nitrogen (BUN). (**L**) Creatinine (Cre). (**M**) Serum calcium (Ca). (**N**) Serum sodium (Na). * *p* < 0.05, ** *p* < 0.01 and *** *p* < 0.001.

Multiple blood parameters of mice in each group were examined, and the results are shown in Figure 6 and Table 1. After being challenged by ETX, white blood cells (WBC), the number of lymphocytes (LYM), basophils ratio (BASO%), number of neutrophils (NEU), neutrophil ratio (NEU%), and number of monocytes (MON) in blood clearly increased, while the LYM ratio (LYM%) decreased (Figure 6B–H), indicating that ETX induced inflammatory response in mice [36]. ETX also led to increased alkaline phosphatase (ALP) (Figure 6I), suggesting liver damage and abnormal liver metabolism [37]. In addition, an abnormal increase in glucose (GLU) (Figure 6G) was presumably because liver damage prevented the degradation of GLU, which is mainly degraded in the liver [38]. A decreased urea nitrogen (BUN) and creatinine (Cre) and increase in serum calcium (Ca) and serum sodium (Na) (Figure 6K–N) indicate that ETX could damage the kidney of mice [39,40,41,42,43], consistent with previous reports that the kidney is one of the main target organs of ETX. However, after the treatment with ZA, these values tended toward normal; mice with the maximum ZA treatment time had blood biochemical parameters similar to the control group. Thus, results indicate that ZA can effectively protect mice from the toxicity of ETX.

### 2.8. ZA Inhibits the Synthesis of Cholesterol and Disrupts Lipid Rafts

**Figure 7:**
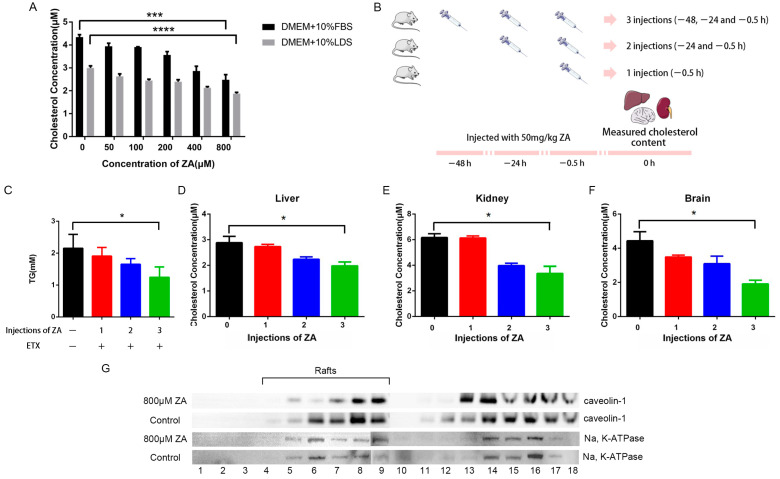
ZA inhibits the synthesis of cholesterol. (**A**) The cholesterol of MDCK cells incubated with ZA for 30 min was measured according to the manufacturer’s instructions using the Amplex red cholesterol assay kit. (**B**) Mice were injected with 50 mg/kg/day ZA for 3 injections (−48, −24, and −0.5 h), 2 injections (−24 and −0.5 h), or 1 injection (−0.5 h) only. (**C**) Triglyceride (TG) levels of blood and cholesterol levels of liver (**D**), kidney (**E**), and brain (**F**) in different groups. (**G**) MDCK cells were incubated with ZA for 30 min, then collected, and the density gradient centrifugation and Western blotting were conducted. * *p* < 0.05, *** *p* < 0.001 and **** *p* < 0.0001.

ZA inhibits cholesterol synthesis, and we hypothesized that ZA reduces the toxic effects of ETX by disrupting the membrane lipid rafts of host cells. We thus measured the cholesterol content of cells treated with a series of concentrations of ZA. As Figure 7A shows, ZA effectively reduced the cholesterol concentration of cells in a dose-dependent manner. For the animal assay, triglyceride (TG) levels of blood and cholesterol content of organs, such as the liver, kidney, and brain, in ZA-treated mice were significantly reduced (Figure 7B–F). To confirm that ZA disrupts lipid rafts, we measured the content of caveolin-1, a marker of lipid rafts on the membranes of MDCK cells. Cells were incubated with ZA for 30 min, then collected, and a density gradient centrifugation was performed. Western blots (Figure 7G) showed that caveolin-1 was recovered in fractions of lower density (fractions 4 to 9) and higher density (fractions 12 to 18). Compared with the control group, the bands of caveolin-1 from MDCK cells treated with ZA were weaker in lower-density fractions. This may indicate that ZA reduced the toxicity of ETX by decreasing the number of lipid rafts present on the membrane of MDCK cells. It is noteworthy that caveolin-1 still exists at a higher density, probably because the association of caveolae with the actin cytoskeleton is not disrupted by the lysis procedure, resulting in the generation of relatively heavy caveolar membranes. The results indicate that ZA decreases lipid rafts on the cell membrane by inhibiting the synthesis of cholesterol and that this more likely protects against the toxicity of ETX in vitro and in vivo.

### 2.9. Other Statin Medicine Inhibits the Toxicity of ETX

**Figure 8:**
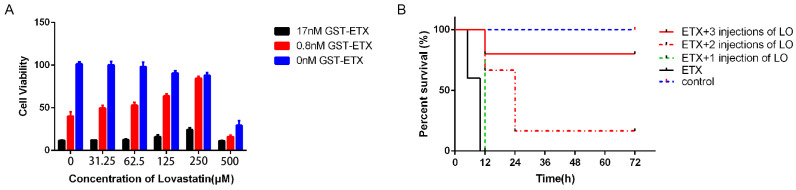
Lovastatin (LO) inhibits the toxicity of ETX. (**A**) MDCK cells were incubated with LO for 30 min, and then exposed to ETX for 1 h. The cytotoxicity of ETX was measured by MTS assay. (**B**) Mice were injected with 6400 ng/kg ETX 30 min after the administration of a first, second, or third dose of LO. Mice in the control group were injected with PBS.

Many approved statins other than ZA can lower cholesterol. We thus considered whether other statin medicines could inhibit the toxicity of ETX. To prove this, we treated MDCK cells with LO prior to exposure to ETX (0.8 nM and 17 nM) and measured cell viability with an MTS assay. LO inhibited ETX toxicity in a dose-dependent manner; the protective rate of cells exposed to 0.8 nM ETX, with a concentration of 250 μM, LO was nearly 100%, and the same concentration of LO increased cell viability under 17 nM ETX from 11% to 23% (Figure 8A). When mice were challenged by 6400 ng/kg ETX, compared with the untreated mice, the survival of mice pretreated with 10 mg/kg LO significantly increased from 0% to 80% (Figure 8B). These results indicated that other statins such as LO can be used to prevent ETX poisoning.

### 2.10. ZA Inhibits the Toxicity of Other Pore-Forming Toxins

**Figure 9:**
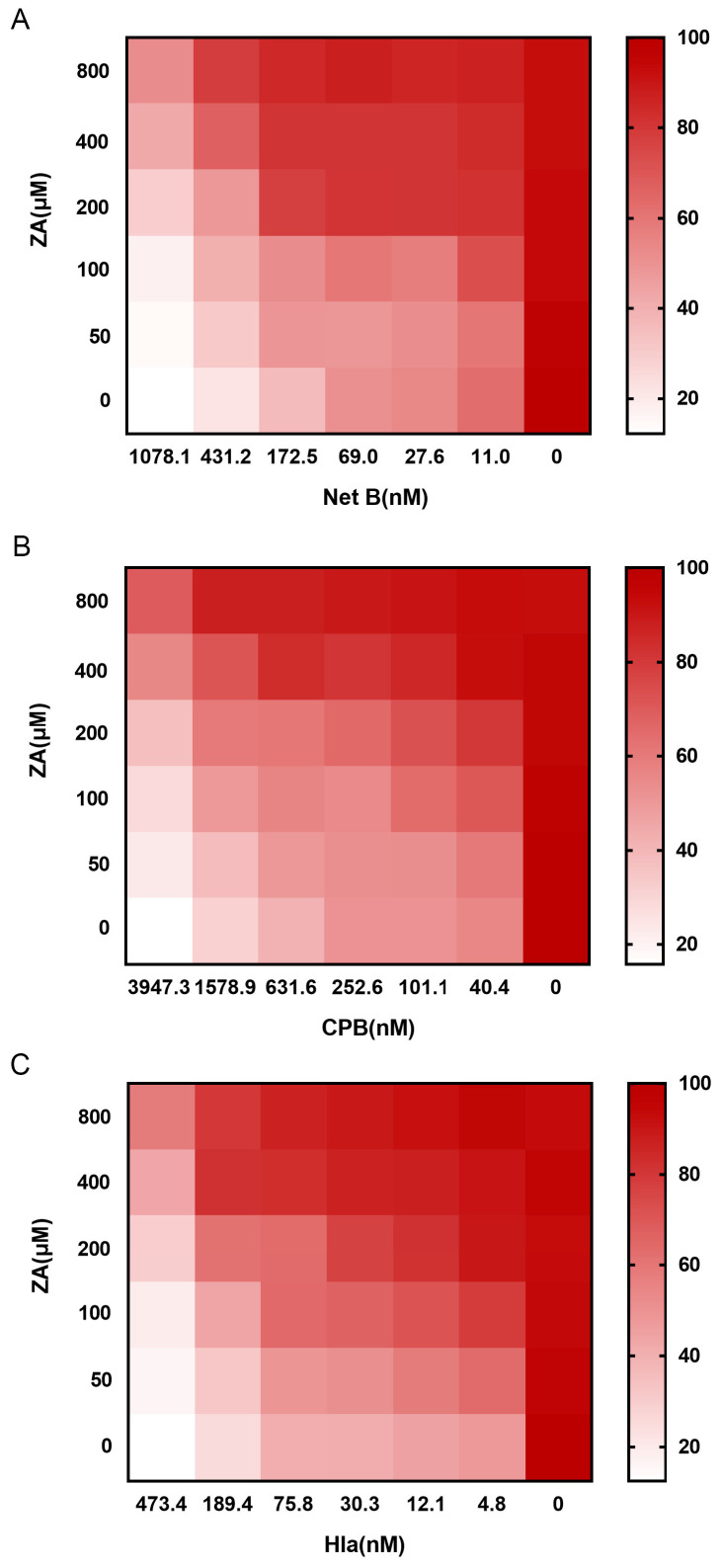
ZA reduces the toxicity of other pore-forming toxins. MDCK cells were incubated with ZA for 30 min, then exposed to *Clostridium perfringens* Net B (Net B) (**A**), *Clostridium perfringens* β-toxin (CPB) (**B**), or *Staphylococcus aureus* α-hemolysin (Hla) (**C**), and observed for 1 h.

We demonstrated that ZA inhibits the toxicity of ETX by disrupting the membrane lipid rafts of host cells. Considering many pore-forming toxins require lipid rafts, we hypothesized that ZA could inhibit the toxicity of other pore-forming toxins. The MTS assay was used to test whether the inhibiting ability of ZA extended to other pore-forming toxins (Hla, CPB, and Net B). We chose MDCK cells for Hla and CPB based on their known sensitivity to these toxins (Figure S7). The CT_50_ dose of Hla to MDCK cells is 10.72 nM, the CT_50_ dose of CPB to MDCK cells is 136.2 nM, and the CT_50_ dose of Net B to MDCK cells is 22.46 nM. The three toxins were used to measure the protective effect of ZA on cells. ZA reduced the toxicity of these toxins in a concentration-dependent manner (Figure 9). The EC_50_ values for ZA was shown in Table 2. Specifically, 800 μM ZA increased the cell viability of MDCK cells from ~50% to ~87% when exposed to the CT_50_ dose of Net B. ZA at the same concentration almost completely inhibited the toxicity of CPB or Hla on MDCK cells. Thus, ZA appears to protect against a wide variety of pore-forming toxins.

## 3. Discussion

ETX is a potent toxin, causing serious zoonotic diseases such as enterotoxaemia. It is also a potential bioterrorism agent. As such, ETX threatens the health of both livestock and humans. However, there are no effective therapies against ETX-induced diseases. Inspired by the fact that the cytotoxicity of ETX is dependent on the integrity of lipid rafts [22], we speculated that statin medicines might act as a medical countermeasure to reduce the toxicity of ETX. In this study, we show that ZA reduces the toxic effects of ETX on MDCK cells and protects mice against ETX in vivo by disrupting the ETX-induced pore-formation in lipid rafts.

Lipid rafts play a central role in many cellular processes, including membrane sorting and trafficking, cell polarization, and signal transduction processes [44]. Several groups of pathogens, bacteria, prions, viruses, and parasites hijack lipid rafts for their purposes [45]. Lipid rafts in cell plasma membranes play a critical role in the life cycle of many viruses such as severe acute respiratory syndrome coronavirus 2 [46]. Lipid rafts are also key to the toxicity of many toxins [22,47,48]. ETX forms heptameric pores within the detergent-insoluble microdomains of MDCK cells and removal of cholesterol by MbCD impairs the complex formation of ETX [13,22]. Our results showed that ZA largely impedes the formation of pores by ETX, consistent with the findings of previous studies. Pore formation by ETX requires the fluidity of putative receptor molecules in the lipid rafts [49]. We suggest that ZA lowers the cholesterol level in lipid rafts and disrupts the lipid rafts of host cells. Cholesterol is thought to serve as a spacer between the hydrocarbon chains of the sphingolipids and to function as a dynamic glue that keeps the raft assembly together [44]. Hence, we suggested that ZA disrupts membrane lipid rafts of host cells by reducing cholesterol levels. It’s reported that cultured cells get most of their cholesterol from the serum in the media, not via de novo synthesis [50]. To exclude the influence of the cholesterol in the medium on the experiment, the MDCK cells were cultured with DMEM supplemented with 10% FBS or 10% LDS. ZA can reduce cholesterol in cultured cells regardless of whether the medium is supplemented with cholesterol or not. In addition, in contrast to lovastatin, which inhibits HMGCR [51], ZA inhibits the synthesis of squalene [52,53], which is located downstream of cholesterol synthesis. Considering ZA can effectively reduce the cholesterol concentration of cells, we speculate that ZA is less likely to protect cells by inhibiting sterols other than cholesterol. It is also possible that ZA inhibits the cytotoxicity of ETX by more than one pathway.

Surprisingly, we found that ZA can increase the influx of Ca^2+^ in MDCK cells. The role of Ca^2+^ influx in the cytotoxicity of ETX is not yet precisely understood. Some studies have shown that Ca^2+^ influx may contribute to the swelling and apoptosis of MDCK cells exposed to ETX [13,31]. However, in our previous study, the activation of Ca^2+^ influx pathways did not always potentiate hemolysis of human erythrocytes, suggesting it may not be an absolute requirement for lysis to occur [32]. Another study found that Ca^2+^ influx may protect the cell from swelling and lysis [35]. ZA alone also induces potent Ca^2+^ influx in MDCK cells (Figure S6) but not cell death (Figure 1), and, thus, ZA-induced Ca^2+^ influx in MDCK cells likely has a positive effect on cells. This may suggest that ZA inhibits the cytotoxicity of ETX by more than one pathway.

ZA protected mice against an ETX challenge. ZA is a blood-cholesterol-lowering agent, which may take hours or days to work in animals. Therefore, in this study, we measured the therapeutic effect of ZA against a high dose of ETX after pre-treating mice with ZA at −48, −24, and −0.5 h prior to the challenge. Results were consistent with the previous hypothesis that the longer mice are pretreated with ZA, the higher their survival rates when challenged with ETX. In addition, ZA is effective in reducing various symptoms of mice caused by ETX including inflammatory response and damage to the kidney and liver.

ZA belongs to the large family of statin medicines, such as atorvastatin, fluvastatin, and lovastatin, many of which have been approved by the FDA for clinical use. Our results indicate that other statins, such as lovastatin, can be used in the treatment of ETX poisoning in mice. Statin medicines are typically cheap and widely available. Developing therapeutic medicine from available statins provides a clear advantage.

Since cholesterol is essential for numerous pore-forming toxins, especially cholesterol-binding toxins, statins provide a good starting point for the treatment of multiple toxins. We show that ZA can inhibit the toxicity of many pore-forming toxins, such as Hla, CPB, and Net B, opening up the possibility of ZA as a promising broad-spectrum candidate to defend against a wide variety of pore-forming toxins.

## 4. Materials and Methods

### 4.1. Recombinant Toxins and Reagents

Horseradish peroxidase (HRP)-conjugated goat anti-mouse IgG (H + L) antibody and anti-His monoclonal antibody were purchased from Abcam (Cambridge, MA, USA). 3-(4,5-dimethylthiazol-2-yl)-5(3-carboxymethoxyphenyl)-2-(4-sulfophenyl)-2H-tetrazolium inner salt (MTS) was purchased from Promega Corporation (Madison, WI, USA). Anti-glutathione S-transferase (GST) monoclonal antibodies were purchased from EARTHOX Life Sciences (Millbrae, CA, USA). Annexin V, annexin-V-binding buffer, and PE anti-human CD235a (glycophorin A) antibodies were purchased from BioLegend (San Diego, CA, USA). DAPI (4′,6-diamidino-2-phenylindole), fluo-4, and propidium iodide were purchased from Sigma (St. Louis, MO, USA). Zaragozic acid A trisodium salt (ZA) was purchased from ChemCruz (Huissen, The Netherlands).

Recombinant toxins, 6×His-tagged ETX, GST-tagged ETX, mScar-ETX (mScarlet fluorescent protein fusion protein), CPB, Hla, and Net B proteins, were expressed and purified as previously described [32,54].

### 4.2. Cell Culture and Animals

MDCK cells were grown and maintained in Dulbecco’s Modified Eagle Medium (DMEM, Gibco, Carlsbad, CA, USA) and supplemented with 10% fetal bovine serum (FBS, Gibco, Carlsbad, CA, USA) or 10% lipid-depleted fetal bovine serum (LDS, VivaCell, Shanghai, China) at 37 °C in an atmosphere of 5% CO_2_. BALB/c mice (6–8 weeks old, 20–25 g body weight) were purchased from Vital River (Beijing, China).

### 4.3. Cytotoxicity Assay

Three recombinant ETX with different tags (Glutathione-S-transferase (GST), 6×His, and red fluorescent protein mScarlet (mScar)) were used in this study. A concentration of 10^5^ MDCK cells/mL was grown to confluence in 96-well plates for 24 h. After 3 washes with PBS, cells were exposed to mScar-ETX, His-ETX, and GST-ETX at 20 levels ranging evenly from 0 (as a control) to 2100 nM (diluted by double volume) and incubated at 37 °C for 1 h. MTS was added to plate wells, and toxicity was estimated by measuring absorbance at 492 nm. The CT_50_ dose of CPB, Hla, or Net B to MDCK cells also were measured by MTS assays. Subsequently, cells were treated with ZA (800, 400, 200, 100, and 50 μM) for 30 min, then incubated with toxins for 1 h. Toxins alone (no ZA) were added to cells as a positive control, and DMEM was added to the cells as a negative control.

Next, MDCK cells grown in 96-well plates for 24 h were treated with different serial concentrations of ZA [0 (as a control), 12.5, 25, 50, 100, 200, 400, and 800 μM] for 30 min at 37 °C. GST-ETX, CPB, Net B, or Hla was then added to the medium, and cells were incubated at 37 °C for 1 h. The cytotoxic activity of pore-forming toxins was measured using the MTS colorimetric assay. In a third experiment, MDCK cells were observed using a Molecular Devices ImageXpress Micro confocal microscope (Molecular Devices, California, CA, USA). Briefly, cells cultured in 96-well plates for 24 h were washed with PBS 3 times, then preincubated with 800 μM ZA. After 30 min, different concentrations of GST-ETX (0, 0.8, and 17 nM) were added to the wells, immediately after which cells were observed under the confocal microscope. Each experiment was performed in triplicate.

### 4.4. Binding of Recombinant Proteins to MDCK Cells

MDCK cells (~10^5^ cells/mL) were seeded in a confocal dish and incubated at 37 °C for 24 h. Cells were then washed with PBS 3 times and incubated with ZA for 30 min, followed by mScar-ETX for 1 h. After being washed again three times with PBS, the cells were stained using DAPI. Samples were observed using a laser confocal scanning microscope (SP8; Leica, Wetzlar, Germany).

### 4.5. Heptameric Oligomerization

To observe the effect of the formation of ETX complexes in MDCK cells, cells were grown to confluence in 150 mm diameter plates and then incubated in the same culture medium with ZA (800 μM or 0 μM as a control) for 30 min at 37 °C. Then, His-ETX (17 and 0.8 nM) was added to the medium, and cells were incubated for 1 h. After incubation, cells were washed 3 times with PBS and scraped off with a rubber policeman into 500 µL of ice-cold lysis buffer (PBS with 1% Triton-X 100) supplemented with 1% protease inhibitor. The lysates were centrifuged at 16,000× *g* for 30 min at 4 °C. Supernatants and pellets were electrophoresed on a 15% polyacrylamide SDS-PAGE gel and electrically transferred to a PVDF membrane (Millipore, Burlington, MA, USA). After blocking with 5% skim milk powder for 1 h, the membrane was incubated with a primary antibody (mouse anti-His monoclonal antibody diluted in PBST as 1:1000) at 4 °C overnight, and then incubated with HRP-conjugated secondary antibodies (goat anti-mouse polyclonal antibodies diluted in PBST as 1:5000) for 2 h. The blots were imaged and analysis of bands using the ImageQuant LAS4000 system (GE Healthcare, Boston, MA, USA).

### 4.6. Pore-forming Assay

MDCK cells were grown on a confocal dish and incubated with or without 800 µM ZA for 30 min, followed by exposure to GST-ETX (17, 0.8, or 0 nM as a control) for 1 h. PI was also added to the culture medium with GST-ETX. After three washes with PBS, cells were stained with DAPI and examined using a laser confocal scanning microscope (SP8; Leica, Wetzlar, Germany). The percentage of PI-positive cells was obtained by dividing the number of PI-positive cells by the total number of cells (PI/DAPI).

### 4.7. LDH Assay

We assumed that increased LDH concentrations reflected pore formation. To test this hypothesis, MDCK cells were incubated with 800 µM ZA for 30 min and then exposed to ETX (17 nM, 0.8 nM, and 0 nM as control) for 60, 45, 30, 15, 10, 5, or 0 min. The leakage of cellular LDH was measured in cell culture supernatants using the LDH-Glo^TM^ cytotoxicity assay kit (Promega Corporation, Madison, WI, USA) according to the manufacturer’s instructions.

### 4.8. Flow Cytometry

MDCK cells were grown on a 6-well plate, preincubated with 800 µM ZA for 30 min, and then incubated with GST-ETX (0.8 or 17 nM) for 1 h at 37 °C. After incubation, cells were washed 3 times with PBS and digested with 0.25% trypsin to get a suspension of cells. For annexin-V-binding studies, 10^5^ cells were analyzed per experimental condition. PI was added to the same suspension. The cells were centrifuged for 10 min at 1000× *g*, resuspended in a solution containing annexin V and annexin V binding buffer, and incubated in the dark for 10 min. The suspension was diluted 5-fold in Ca^2+^-containing saline, and then analyzed on a FACSaria flow cytometer (Becton Dickinson and Company, New Jersey, NJ, USA) with excitation at 488/535 nm and emission at 520/615 nm. All experiments were conducted at 37 °C.

The intracellular Ca^2+^ concentration was also measured on a FACSaria flow cytometer. MDCK cells were preincubated with 800 µM ZA for 30 min and then digested with 0.25% trypsin to get single-cell suspensions. A suspension of MDCK cells (~10^6^ cells/mL) was incubated for 25 min at fluo-4 AM (5 μM) at 37 °C, washed once with PBS, and then centrifuged at 1000× *g* for 10 min at room temperature. After the addition of ETX (0.8 and 17 nM) in Ca^2+^-containing saline and incubation for 10 min, the cells were measured in a FACSaria flow cytometer with excitation at 488 nm and emission at 520 nm.

### 4.9. Animal Experiments

To learn whether ZA can inhibit the toxicity of ETX in vivo, we analyzed its effect in a murine model. BALB/c mice approximately 6 weeks old were injected with 0.1 mL of ZA (50 mg/kg/day) 1 (−0.5 h), 2 (−24 and −0.5 h), or 3 times (−48, −24, and −0.5 h) before being challenged with GST-ETX (6400 ng/kg) at time 0. As a control, 1 group of mice was injected with 0.1 mL of PBS with −48, −24, and −0.5 h. Mice were monitored for 3 days, and survival was recorded. Finally, blood was taken from the hearts of all mice for biochemical analysis, and the samples of organs dissected from mice were fixed in 4% formaldehyde for 24–48 h.

Samples from dissected mouse organs were dehydrated using ethanol solutions of increasing concentration and xylene solution and then embedded in paraffin. The paraffin-embedded tissue was sliced into 5-µm-thick sections. Sections were heated at 63 °C for 2 h, followed by dewaxing of xylene and decreasing ethanol solutions. Sections were stained with hematoxylin and eosin (H & E). Photographs of the sections were taken using a bright field microscope with a digital camera (Olympus IX71, Tokyo, Japan).

### 4.10. Density Gradient Centrifugation

MDCK cells were plated onto D150 plates. After 24 h, the plates of cells were washed with PBS and pre-incubated with 800 μM ZA for 30 min. Cells were then washed and scraped into base buffer (20 mM Tris-HCl, 250 mM sucrose, 1 mM CaCl_2,_ and 1 mM MgCl_2_, to which was added 1% protease inhibitors; pH = 7.8). Cells were pelleted by centrifugation for 2 min at 250× *g* and resuspended in 1 mL of base buffer. The cells were then lysed by ultrasonication for 1 min, and lysates were centrifuged at 12,000× *g* for 10 min. The supernatant was collected and transferred to a separate tube. The sediment was resuspended with 1 mL base buffer and ultrasonicated for 1 mL. After centrifugation at 12,000× *g* for 10 min, the second supernatant was combined with the first. An equal volume (2 mL) of base buffer containing 50% OptiPrep (Stemcell, Vancouver, VAN, Canada) was added to the combined postnuclear supernatants and placed in the bottom of a 12 mL centrifuge tube. An 8 mL gradient of 0% to 20% OptiPrep in base buffer was poured on top of the lysate. Gradients were centrifuged for 90 min at 52,000× *g* using an SW-41i rotor in a Beckman ultracentrifuge. Gradients were fractionated into 0.67 mL fractions, and the distribution of proteins was assessed by Western blotting.

### 4.11. Cholesterol Assay

For measurement of cholesterol, MDCK cells were seeded at 10^5^ cells/mL in complete culture medium (containing 10% FBS or LDS) in 96-well plates. After 24 h, media was replaced with ZA (800, 400, 200, 100, 50, or 0 μM as control) for 30 min. Total cellular cholesterol was measured using the Amplex red cholesterol assay kit (Invitrogen, Carlsbad, CA, USA) according to the manufacturer’s instructions. For the animal assay, mice were injected with ZA 1 to 3 times (at −48, −24, and −0.5 h; Figure 7A). After 30 min on the last day, liver, kidney, and brain tissue were dissected from mice and ground. Cholesterol content in organs was measured using the Amplex red cholesterol assay kit.

### 4.12. Statistical Analysis

Flow cytometry data were analyzed using analysis of variance (ANOVA) and Student’s paired *t*-tests. *p* < 0.05 was used as the criterion for statistically significant differences between groups.

## 5. Conclusions

In conclusion, we found that ZA significantly reduced the toxicity of ETX to MDCK cells and mice. Subsequently, we demonstrated that ZA did so by disturbing the associated pore formation and not affecting binding to host cells. In addition, ZA decreased the exposure of PS on the plasma membrane and promoted the Ca^2+^ influx of the cells, which likely contributed to the attenuation of ETX cytotoxicity. ZA inhibits the synthesis of cholesterol and further disrupts membrane lipid rafts of cells. Excitingly, ZA protected mice against ETX. ZA also inhibited the toxicity of other pore-forming toxins and thus may prove to be a broad-spectrum therapeutic medicine. Furthermore, other statins, such as LO, also can reduce the toxicity of ETX. These findings indicate that statin medicines are potential candidates for preventing and treating multiple toxin-induced diseases.

## Figures and Tables

**Table 1:** Parameters of blood biochemical indexes and blood routine examination in mice.

Groups	Control	1 Injection of ZA	2 Injections of ZA	3 Injections of ZA	Challenged with ETX
WBC (10^9^/L)	1.53 ± 0.60	2.39 ± 1.10	2.07 ± 0.41	1.78 ± 0.80	3.42 ± 0.97
LYM (10^9^/L)	1.12 ± 0.37	1.62 ± 0.22	1.51 ± 0.26	1.13 ± 0.47	1.94 ± 0.59
LYM (%)	74.00 ± 7.16	64.20 ± 2.54	72.80 ± 2.04	72.62 ± 7.13	46.90 ± 9.85
BASO (%)	0.85 ± 0.23	1.50 ± 0.88	0.77 ± 0.41	0.50 ± 0.11	1.82 ± 0.77
NEU (10^9^/L)	0.33 ± 0.23	0.47 ± 0.12	0.41 ± 0.14	0.56 ± 0.30	1.54 ± 0.49
NEU (%)	19.60 ± 7.19	30.37 ± 3.34	24.73 ± 2.13	19.76 ± 4.08	46.21 ± 11.82
MON (10^9^/L)	0.04 ± 0.03	0.05 ± 0.03	0.04 ± 0.01	0.03 ± 0.01	0.10 ± 0.03
ALP (U/L)	2.39 ± 0.51	1.77 ± 0.46	1.86 ± 0.53	1.79 ± 0.38	3.17 ± 0.27
GLU (mM)	7.79 ± 1.33	8.01 ± 1.51	7.87 ± 1.31	7.72 ± 1.72	13.51 ± 2.28
BUN (mM)	11.81 ± 1.28	12.38 ± 0.95	14.52 ± 0.87	13.63 ± 1.26	6.77 ± 1.70
Cre (μM)	7.14 ± 0.72	6.78 ± 1.61	7.38 ± 1.59	7.84 ± 1.45	5.75 ± 0.70
Ca (mM)	0.076 ± 0.011	0.075 ± 0.005	0.086 ± 0.01	0.075 ± 0.005	0.100 ± 0.01
Na (mM)	230.60 ± 3.28	225.00 ± 6.21	242.50 ± 9.19	235.20 ± 7.50	265.50 ± 19.05

**Table 2:** The EC_50_ (concentration for 50% of the maximal effect) value for ZA against pore-forming toxins.

Toxin	EC_50_ (μM)
ETX	151.80
Net B	72.42
Hla	38.20
CPB	430.5

## Data Availability

Data from this study are available within the article and its Supplementary Information files or from the corresponding author upon reasonable request.

## Supplementary Materials

The supporting information can be downloaded at: https://www.mdpi.com/article/10.3390/ijms24065414/s1.
