# Supplementary material for: Statins as Potential Preventative Treatment of ETX and Multiple Pore-Forming Toxin-Induced Diseases
Source: Int J Mol Sci. 2023 Mar 12;24(6):5414. doi: 10.3390/ijms24065414 (PMC10048941; doi:10.3390/ijms24065414)
Supplement: Supplementary file 1 [file ijms-24-05414-s001.zip › ijms-2188846-supplementary.pdf]

Supplementary data

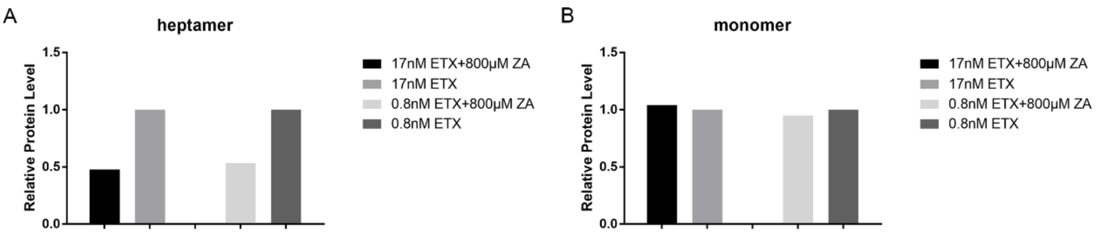

Supplementary Figure S1. ZA reduces the formation of ETX heptamers in MDCK cells. The amount of heptamer **(A)** and monomer **(B)** of Figure 3A were quantitated.

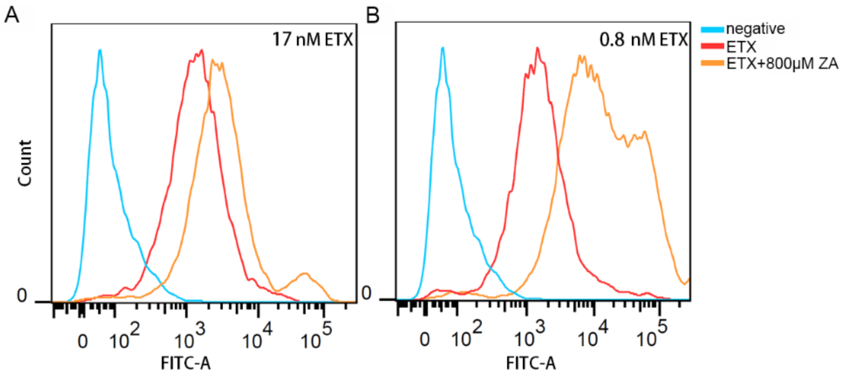

Supplementary Figure S2. ZA strengthens the Ca^{2+} influx in ETX-treated MDCK cells. This figure is the frequency curve of Figure 4B.

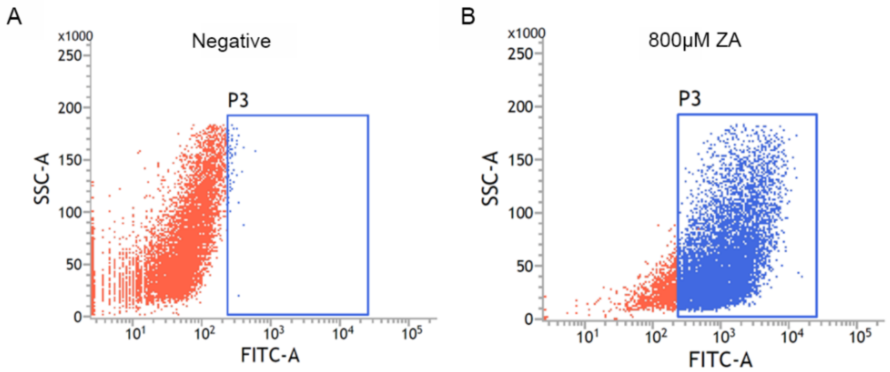

Supplementary Figure S3. ZA alone induces Ca^{2+} influx in MDCK cells. MDCK cells were preincubated in DMEM medium with (B) or without (A) 800 μM ZA for 30 min and then digested with 0.25% trypsin to get single-cell suspensions. A suspension of MDCK cells (~10^6 cells/mL) were incubated for 25 min in fluo-4 AM (5 μM) at 37°C, washed once with a Ca^{2+}-containing saline, and incubation for 10 min. Then the cells were applied in a FACSaria flow cytometer via 488-nm excitation and 520-nm emission.

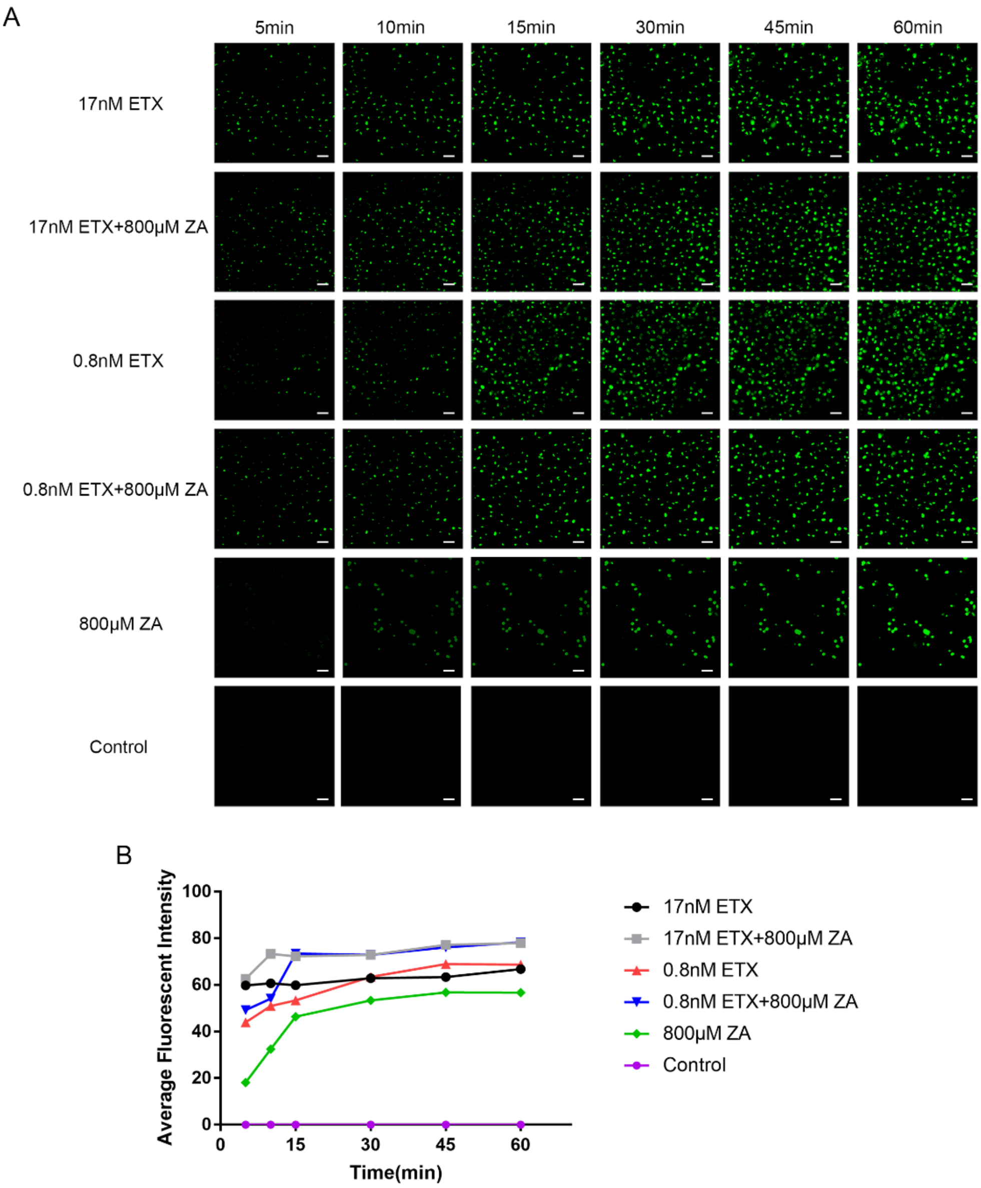

Supplementary Figure S4. ZA induces Ca^{2+} influx in MDCK cells. (A) MDCK cells were preincubated in DMEM medium with or without 800 μM ZA for 30 min and then exposed to a Ca^{2+}-containing saline including ETX (17, 0.8 and 0 nM) and fluo-4 AM (5 μM) at 37°C. Then the cells were observed using a laser confocal scanning microscope (SP8; Leica, Wetzlar, Germany). **(B)** The average fluorescent intensity was measured using Image-Pro Plus.

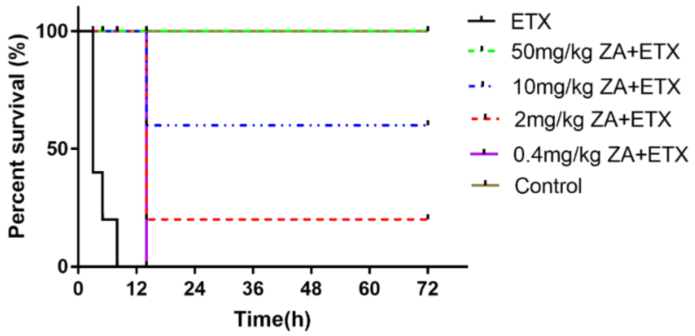

Supplementary Figure S5. ZA can prevent the toxin-induced death of mice. Mice were given an intraperitoneal injection of ZA (50, 10, 2 and 0.4 mg/kg/day) for 3 injections (-48, -24 and -0.5 h) and then challenged with ETX (6400 ng/kg) on time 0 and injected with PBS as control.

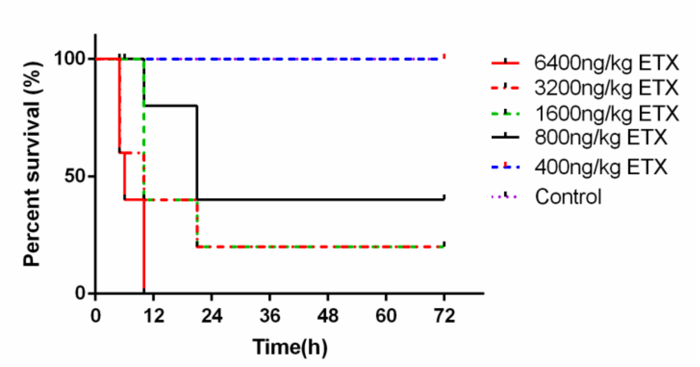

Supplementary Figure S6. Determination of absolute lethal dose of ETX to mice. Mice were challenged with 0.1 ml ETX (6400, 3200, 1600, 800 and 400 ng/kg) and injected with PBS as control. Then, observed for 72 h and statistical the survival rate.

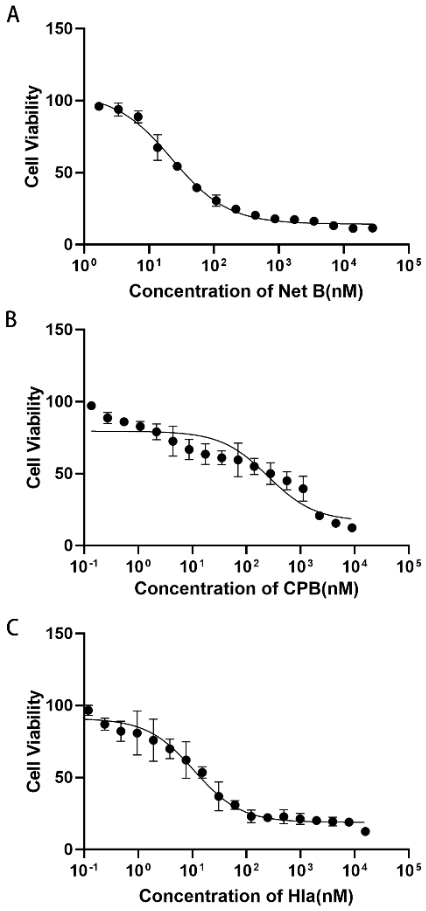

Supplementary Figure S7. Cytotoxicity of pore-forming toxins toward MDCK cell line. MDCK cells were incubated with ZA for 30 min, then exposed to Net B **(A)**, CPB **(B)** or Hla **(C)** and observed for 72 h.
